# Intestinal carriage of *Campylobacter jejuni *and *Campylobacter coli *among cattle from South-western Norway and comparative genotyping of bovine and human isolates by amplified-fragment length polymorphism

**DOI:** 10.1186/1751-0147-48-4

**Published:** 2006-06-06

**Authors:** G Johnsen, K Zimmerman, B-A Lindstedt, T Vardund, H Herikstad, G Kapperud

**Affiliations:** 1 National Veterinary Institute, Oslo, Norway; 2 The Norwegian Food Control Authority, Sandnes, Norway; 3 Norwegian Institute of Public Health, Oslo, Norway; 4 The Norwegian School of Veterinary Science, Oslo, Norway

## Abstract

In a survey conducted in 1999–2001, the carriage of thermotolerant *Campylobacter*s in cattle was investigated, and the genetic diversity of *C. jejuni *within one herd was examined and compared with human isolates. *C. jejuni*, *C. coli *and other thermotolerant *Campylobacter *spp. were isolated from intestinal contents from 26%, 3% and 2% of 804 cattle, respectively. The carriage rate was higher in calves (46%) than in adults (29%). Twenty-nine *C. jejuni *isolates from one herd and 31 human isolates from the study area were genotyped with amplified-fragment length polymorphism (AFLP). Eighty-three % of the bovine isolates fell into three distinct clusters with 95–100% similarity, persistent in the herd for 5–10 months. Among human isolates, 58% showed >90% similarity with bovine isolates. The results show that cattle are a significant and stable reservoir for *C. jejuni *in the study area. Transmission between individuals within the herd may be sufficient to maintain a steady *C. jejuni *population independent of environmental influx. The results of this study have provided new information on *C. jejuni *and *C. coli *transmission, and also on the carriage in cattle, genotypes stability and similarity between bovine and human isolates.

## Introduction

During the last 30 years, campylobacteriosis has emerged as an important food-borne bacterial zoonosis causing acute diarrhoea in humans at low infection doses [1-4]. In many developed countries, the disease is currently the most frequently reported bacterial enteric infection in humans [1,3,4]. Since the beginning of the 1990's, a substantial increase has been observed in several countries [3]. In Norway, the incidence of campylobacteriosis doubled from 1994 to 1999, according to the Norwegian Surveillance System for Communicable Diseases [5]. Case control studies of sporadic cases, including a recent one from Norway, have identified a number of risk factors [1,3,6]. The most frequently reported factors are consumption of poultry meat, contact with animals, including farm animals as cattle, poultry and sheep, drinking un-disinfected water, barbequing, and drinking un-pasteurized milk [1,3,4]. The sources most frequently incriminated in outbreaks of campylobacteriosis are drinking water, un-pasteurized milk and poultry [1,3,4,7].

Thermotolerant *Campylobacter *spp. are widespread in nature where the principal reservoirs are the alimentary tracts of wild and domesticated mammals and birds [1,2,7]. Healthy cattle are a recognized reservoir for *Campylobacter*. Reported frequencies range from 0.8 to 100 % in faecal material from adult animals [8,9]. Although consumption of beef products has rarely been identified as a risk factor, the importance of cattle as a reservoir for human infection is highlighted by the following observations: 1) raw milk as a source in numerous outbreaks, and 2) identification of cattle contacts and raw milk consumption as risk factors in epidemiological studies. However, the relative contribution of these factors to overall disease burden is not known [2,3,10].

Management of cattle in Norway is distinctively different from central parts of Europe, Canada and the United States. The majority of Norwegian cattle belong to one stock, the Norwegian red cattle (NFR). Farms are usually small, housing in average around 30 animals, and raising both dairy and meat cattle, which are fed with the same type of feed. Both slaughtering and calving are evenly distributed throughout the year, and there is no tradition for special fattening calves. The animals spend about eight months indoor, and when they are out, sharing of grazing fields with herds from other farms and frequently shifting of fields are common.

A number of methods have been developed to investigate genetic diversity among *Campylobacter*s in order to trace sources of infection [11,12]. The methods differ in their taxonomic range, discriminatory power, reproducibility and ease of interpretation and standardization. Amplified-fragment length polymorphism (AFLP) genotyping is a comparatively rapid method based on PCR and capillary electrophoresis, which combines universal applicability with high discriminatory power [13].

In the present investigation, we assessed the occurrence of *C. jejuni *and *C. coli *in cattle from South-western Norway, an area with one of the highest incidence rates of human campylobacteriosis in the country [5]. The aims of the study were to: 1) investigate the frequency of *C. jejuni *and *C. coli *in cattle from the study area, 2) examine the genetic diversity among *C. jejuni *isolates from one of the herds using AFLP, and 3) compare the AFLP profiles of isolates from cattle and human patients from the study area.

## Materials and methods

### Study population and sampling plan

We examined a total of 804 samples of intestinal contents from cattle representing 333 herds from 16 municipalities in Rogaland County (n = 764) and 8 municipalities in Vest-Agder County (n = 40), South-western Norway. Approximately one-sixth of all cattle produced in Norway originate from this area. The animals were sampled at two abattoirs, which slaughter approximately one third of all cattle in the counties under study. The samplings were performed by the staff at the official meat control. However, there were some deviations in the intended sampling schedule, because the veterinarians responsible for sampling could be absent due to other duties. A pilot study of 88 animals from 74 different herds was conducted during the 6-weeks period from 13 April through 27 May 1999 at the greater of the two abattoirs. One to two days each week, samples from ten animals were collected evenly throughout the day. Since the results of the pilot study showed that the frequency was sufficient to warrant further investigation, we conducted an extended survey at both abattoirs during the 13-months period from January 2000 through January 2001. Each abattoir was visited once a week, and at each visit, samples were taken evenly throughout the day from five to seven animals. A total of 716 animals representing 277 herds were investigated.

### Sample collection

From each animal, approximately 5 grams of intestinal content were collected aseptically from the descending colon immediately after slaughter. Samples were placed in 7.5 ml Cary Blair transport medium (CM0519, Oxoid Ltd., Basingstoke, UK), mixed, and transported in a thermal bag to the laboratory where samples were stored in a refrigerator until cultivation within 24 hours after collection.

### Cultivation of samples

One gram of each sample was transferred to a tube containing 9.0 ml Preston selective enrichment broth consisting of nutrient broth (Oxoid CM0067) supplemented with 5% lysed horse blood (Oxoid SR0084) and polymyxin B, rifampicin, trimethoprim and cycloheximide (Oxoide SR0117). Tubes were incubated for 24 h at 42.0 ± 0.5°C in micro-aerobic atmosphere (CampyGen, CN0025; Oxoid Ltd.). Ten μl of each enrichment culture were plated on modified charcoal cefoperazone desoxycholate agar (m-CCDA) with amphotericin B (CM0739 and SR0155; Oxoid Ltd.) and incubated 48 h at 42.0 ± 0.5°C in micro-aerobic atmosphere. From each plate, one colony with characteristic morphology was examined by phase-contrast microscopy. Further confirmation was performed with the 2810 AccuProbe *Campylobacter *culture identification test kit using the AccuLDR luminometer (Gene-Probe Inc., San Diego, USA), a rapid DNA probe test, which utilizes the technique of nucleic acid hybridization for the identification of thermotolerant *Campylobacter *spp. Isolates identified as thermotolerant *Campylobacter *spp. were further tested for hydrolysis of hippurate and indoxyl acetate according to standard procedures, which formed the basis for species differentiation [7].

### Human clinical isolates

A total of 31 strains of *Campylobacter jejuni *isolated from human patients were included in the study. The isolates, sampled by the Department of Medical Microbiology, Stavanger University Hospital during a case control study (*Kapperud et al. *2003), included all domestically acquired campylobacteriosis cases in Rogaland County from January throughout June 2000. The isolates were obtained from the strain collection at the Norwegian Institute of Public Health.

### AFLP genotyping

DNA was extracted in a bio robot (EZ-1; Qiagen Instruments AG, Hilden, Germany), according to the manufacturer's instructions. The AFLP analysis was performed as detailed by *Lindstedt et al. *(2000), using the restriction enzymes *Bgl*II and *Mfe*I (New England Biolabs, Beverly, MA, USA). Calculation of similarities between AFLP patterns, cluster analyses, and generation of dendrograms was performed in the computer program GelCompar II (Applied Maths, Kortrijk, Belgium) as described by *Lindstedt et al. *(2000). Based on knowledge of outbreak strains and the reproducibility of the method designated all strains within a window of similarity of between 95 and 100% homology as being identical.

### Data analysis

The data were analysed using the statistical package Intercooled Stata for Windows 9.0 (StataCorp LP, Collage Station, Texas, USA). Due to the lack of random sampling, we were reluctant to undertake any advanced statistical analyses. Frequencies with confidence intervals were calculated using the proportion command in Stata, and age groups were compared using the chi-square test.

## Results

### Occurrence

*C. jejuni *and *C. coli *were isolated from 208 (26%, 95% CI: 23–29%) and 22 (3%, 95% CI: 2–4%) of 804 cattle examined, respectively. In addition 13 (2%, 95% CI: 1–3%) of the isolates were other thermotolerant *Campylobacter *spp. not hydrolyzing hippurate and indoxyl acetate, and these were not classified at the species level. In total, thermotolerant *Campylobacter*s were isolated from 30% (95% CI: 27–34%) of the cattle.

The frequency of thermotolerant *Campylobacter*s among adult cattle (n = 715) and among calves between the age of 1.5 and 9 months (n = 74) were 28.5% and 46.0%, respectively. Chi squared analyses adjusting for month show that there were significant differences in frequency between the two age groups (p = 0.0002). The frequency of carrier rates in different months ranged from 0% in January 2000 to 47% in July 2000 (median, 30%).

### AFLP genotyping

**Figure 1:**
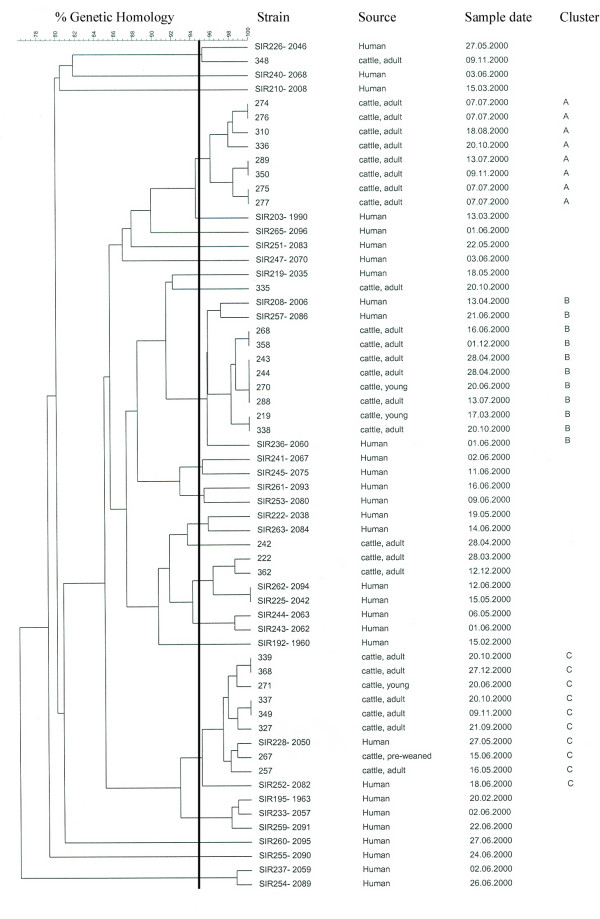
Dendrogram based on AFLP fragment patterns of 60 *Campylobacter jejuni *isolates, 29 from intestinal material from one cattle herd and 31 clinical isolates from human patients in the study area during the study period, with strain identity, source of isolation, isolation date and AFLP profile identity. The vertical line marks 95% similarity.

Of the 333 herds included in the study, five were represented by more than 20 animals and only one (herd HH; n = 119) with more than 40 animals. Cattle in herd HH was regarded representative for the area based on management routines, which included uniform breeding and feeding, common grazing fields and frequent trading between herds. AFLP genotyping was used to differentiate 60 *C. jejuni *isolates, comprising 29 of 32 *C. jejuni *isolates from herd HH (three were lost during freeze storage) and 31 clinical isolates obtained from human patients in the study area during 2000. A total of 19 distinct AFLP profiles were detected among the bovine isolates, while the human isolates showed 30 profiles. All isolates grouped within a window of similarity of 80% (Figure 1). The majority of the bovine isolates (83%, n = 24) fell into three clusters, each containing eight isolates with 95–100% similarity (clusters A, B and C in Figure 1). The genetic distance between these three clusters were approximately 85%. All isolates in cluster A were from animals slaughtered in the period July 7 to November 9, while the cluster B isolates were from March 17 to December 1, and cluster C comprised isolates from May 16 to December 27. Hence, the dates of slaughter for the animals from which the strains were isolated overlapped considerably, and there was no clear difference in the seasonal distribution of the clusters. The remaining five bovine isolates were scattered in the dendrogram. Among the human strains, 18 (58%) showed more than 90% similarity with one or more bovine isolate. At 95% similarity, three human isolates fell into cluster B, and two human strains grouped together with the eight bovine isolates in cluster C.

## Discussion

In the present study, we isolated *C. jejuni *and *C. coli *from about one-third of healthy cattle from South-western Norway. This is higher than reported from Denmark [14] and Sweden [15], but lower than what is found in the UK and Ireland [9].

The higher carriage rate found in calves than in adult cattle, are supported by results from other studies [1,9,14,16,17]. Similar age distributions are also observed among other animals and humans [3,4,18,19]. Whether this is due to acquired immunity among adults or more frequent exposure in young individuals is not known [9,18].

We did not find any significant seasonal difference. This would not have been unexpected, since a pronounced summer peak is observed among broiler chickens as well as in humans in Norway [5,20]. This indicates that cattle herds are able to maintain a stable *Campylobacter *population by transmission within the herd. AFLP genotyping of isolates from one of the herds indicates that three distinct *C. jejuni *clones, each with 95–100% internal similarity, were present through 5–10 months of the study period. Transmission between individuals within the herd is probably sufficient to maintain a steady population of *C. jejuni *without environmental influx. The duration of colonization in individual animals was not determined in our study, but previous investigations have shown that cattle may shed large quantities of *Campylobacter*s for up to 112 days [8].

Our finding that some of the human clinical isolates from the study area showed close genetic similarity with bovine strains should be interpreted with caution. Direct transmission from cattle to humans cannot be completely excluded, though it is more likely that both species were infected from a common source, most notably through water. Water may constitute the common reservoir linking infections in humans and animals [3]. Recent results indicating a symbiotic relation between *Campylobacter *and aquatic amoebae may support this hypothesis [21]. Drinking un-disinfected water was the leading risk factor for human campylobacteriosis in a Norwegian case-control study [6], occupational exposure to cattle and other farm animals was also identified as an independent risk, but beef consumption was not. Consumption of un-pasteurized milk is uncommon in Norway, though a few outbreaks related to raw milk has been reported [22,23].

The study contributed with less information than expected because of deviations in the intended sampling schedule. The culturing methods used in this study select for thermo-tolerant *Campylobacter *species [24]. This may be the reason why mainly *C. jejuni *was isolated. Selective enrichment was used for all samples and may contribute to skewed data. It must also be taken into consideration that only a finite number of colonies were isolated and typed, a limitation allowing the most predominant clones to be isolated. The cattle isolates included in the AFLP typing originated from only one herd, including all *Campylobacter *isolated from this herd. Due to this limitation, it is difficult to imply associations between human and cattle isolates in general.

## Conclusion

The results show that cattle are a significant and stable reservoir for *C. jejuni *in the study area. In one of the herds, three distinct genetic clones were present over several months. The genetic similarity between human and bovine isolates may reflect infection from a common source. The highest carriage rates were found among calves versus adults.

## Sammendrag

*Forekomst av *Campylobacter jejuni *og *Campylobacter coli *i tarm fra storfe fra sørvest Norge og genotypisk sammenligning av bovine og humane isolat ved metoden AFLP*

Forekomst av termotolerante *Campylobacter spp*. hos storfe ble undersøkt i en kartlegging utført i 1999–2001, og den genetiske diversiteten hos *C. jejuni *innen en besetning ble undersøkt og sammenlignet med humane isolat. *C. jejuni*, *C. coli *og andre termotolerante *Campylobacter spp*. ble isolert fra tarminnhold hos henholdsvis 26%, 3% og 2% av 804 storfe. Frekvensen var større hos kalver (46%) enn hos voksne dyr (29%). Tjueni *C. jejuni*-isolat fra en besetning og 31 humane isolat fra samme område ble karakterisert ved den genetiske fingeravtrykksmetoden AFLP. Av storfeisolatene havnet 83% i tre distinkte grupper med 95–100% likhet, vedvarende i besetningen i 5–10 måneder. Av de humane isolatene var 58% mer enn 90% lik storfeisolatene. Resultatene viser at storfe er et betydelig og stabilt reservoar for *C. jejuni *i området. Overføring mellom individer innen en besetning ser ut til å være tilstrekkelig til å opprettholde en stabil *C. jejuni*-populasjon uavhengig av tilførsel fra omgivelsene. Resultatene har gitt informasjon om smitteveier for *C. jejuni *og *C. coli *med økt kunnskap om nivå hos storfe, stabiliteten til genotypene og likhet mellom humanisolat og storfeisolat.
